# *N*-(4-Hydroxy­pheneth­yl)acetamide

**DOI:** 10.1107/S1600536809025409

**Published:** 2009-07-08

**Authors:** Bo Wang, Yun Chai, Peizhen Tao, Mingliang Liu

**Affiliations:** a Institute of Medicinal Biotechnology, Chinese Academy of Medical Sciences, and Peking Union Medical College, Beijing 100050, People’s Republic of China

## Abstract

In the title compound, C_10_H_13_NO_2_, the occurrence of inter­molecular N—H⋯O and O—H⋯O hydrogen bonds between the hydr­oxy and acetamido groups results in the formation of tetra­mers with an *R*_4_^4^(25) graph-set motif. These tetra­mers are further assembled, building up a corrugated sheet parallel to (001).

## Related literature

For the biological activity of *N*-(4-hydroxypheneth­yl)acetamide, see: Garcez *et al.* (2000 ▶); Montedoro *et al.* (1993 ▶). For related structures, see: Chai *et al.* (2009 ▶); Song *et al.* (2008 ▶). For bond-length data, see: Allen *et al.* (1987 ▶). For hydrogen-bond motifs, see: Bernstein *et al.* (1995 ▶); Etter (1990 ▶)
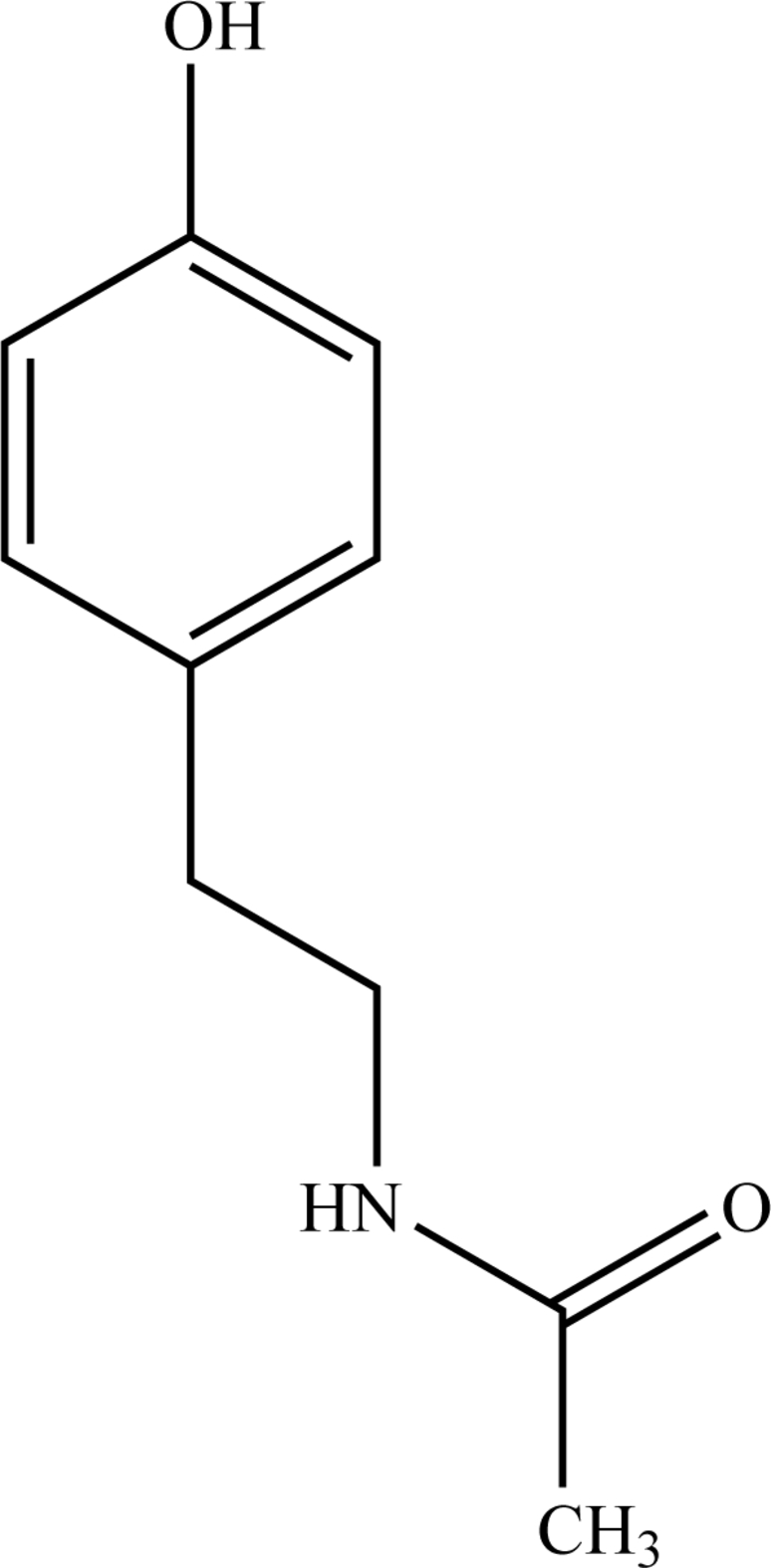


## Experimental

### 

#### Crystal data

C_10_H_13_NO_2_
*M* *_r_* = 179.21
Monoclinic, 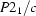

*a* = 9.9206 (13) Å
*b* = 8.7861 (11) Å
*c* = 11.4943 (16) Å
β = 102.9980 (10)°
*V* = 976.2 (2) Å^3^
*Z* = 4
Mo *K*α radiation
μ = 0.09 mm^−1^
*T* = 298 K
0.43 × 0.38 × 0.20 mm

#### Data collection

Bruker SMART CCD diffractometer
Absorption correction: multi-scan (*SADABS*; Sheldrick, 2008*a* ▶) *T*_min_ = 0.961, *T*_max_ = 0.978
4753 measured reflections
1713 independent reflections
1196 reflections with *I* > 2σ(*I*)
*R*_int_ = 0.027

#### Refinement

*R*[*F* ^2^ > 2σ(*F* ^2^)] = 0.037
*wR*(*F* ^2^) = 0.100
*S* = 1.05
1713 reflections
119 parameters
H-atom parameters constrained
Δρ_max_ = 0.16 e Å^−3^
Δρ_min_ = −0.14 e Å^−3^

### 

Data collection: *SMART* (Bruker, 1998 ▶); cell refinement: *SAINT* (Bruker, 1999 ▶); data reduction: *SAINT*; program(s) used to solve structure: *SHELXS97* (Sheldrick, 2008*b* ▶); program(s) used to refine structure: *SHELXL97* (Sheldrick, 2008*b* ▶); molecular graphics: *ORTEPIII* (Burnett & Johnson, 1996 ▶), *ORTEP-3 for Windows* (Farrugia, 1997 ▶) and *CAMERON* (Pearce *et al.*, 2000 ▶); software used to prepare material for publication: *SHELXTL* (Sheldrick, 2008*b* ▶).

## Supplementary Material

Crystal structure: contains datablocks global, I. DOI: 10.1107/S1600536809025409/dn2469sup1.cif

Structure factors: contains datablocks I. DOI: 10.1107/S1600536809025409/dn2469Isup2.hkl

Additional supplementary materials:  crystallographic information; 3D view; checkCIF report

## Figures and Tables

**Table 1:** Hydrogen-bond geometry (Å, °)

*D*—H⋯*A*	*D*—H	H⋯*A*	*D*⋯*A*	*D*—H⋯*A*
N1—H1⋯O1^i^	0.86	2.08	2.9048 (18)	161
O1—H1*A*⋯O2^ii^	0.82	1.83	2.6464 (17)	174

Symmetry codes: (i) 
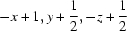
; (ii) 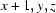.

**Fig. 1.**
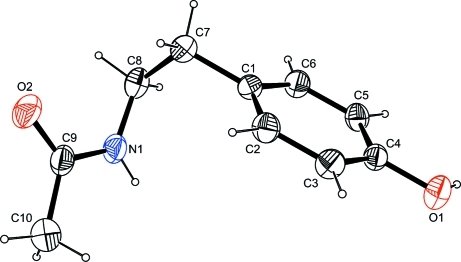
Molecular structure of (I) with the atos labeling scheme. Ellipsoids are drawn at the 30% probability level. H atoms are rpresented as small spheres of arbitrary radii.

**Fig. 2.**
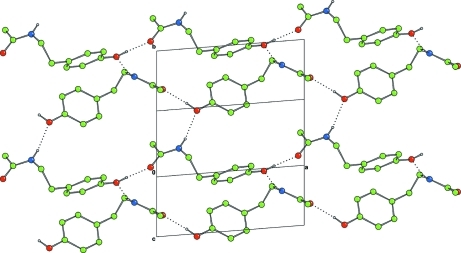
Partial packing view showing the corrugated sheet and the R44(25) graph set motif.

## supplementary crystallographic information

### Comment

*N*-(4-Hydroxyphenethyl)acetamide displays various biological activities (Garcez *et al.*, 2000; Montedoro *et al.*, 1993; Allen *et al.*, 1987), we report herein the crystal structure of the title compound.

In the molecule of the title compound (Fig 1), all of the bond lengths and angles are within normal ranges. the occurrence of intermolecular N–H···O and O–H···O hydrogen bonds between the hydroxy and acetamido groups results in the formation of tetramers with a *R*_4_^4^(25) graph set motif (Etter, 1990; Bernstein *et al.*, 1995). These tetrameric motifs are further assemble to build up a corrugated sheet parallel to the (0 0 1) plane (Table 1, Fig. 2).

The bond lengths and bond angles agree with the values observed in related structures (Chai *et al.*, 2009; Song *et al.*, 2008).

### Experimental

The title compound was separated from fermentation liquor as a white solid. Single crystals suitable for X-ray analysis were obtained by slow evaporation of a methanol/water solution(2:1 *v*/*v*). ^1^H NMR (DMSO-d_6_, δ): 9.14 (1*H*, s, OH), 7.84 (1*H*, br, NH), 6.96 (2*H*, d, PhH_2_), 6.66 (2*H*, d, PhH_2_), 3.14–3.19 (2*H*, m, CH_2_), 2.49–2.57 (2*H*, m, CH_2_), 1.77 (3*H*, s, CH_3_). MS(FAB, m/*z*): 180 (*M*+H)^+^.

### Refinement

All H atom were placed at calculated positions, with C—H = 0.96–0.97 Å and N—H = 0.86 Å, and were included in the final cycles of refinement using a riding model, with *U*_iso_(H) = 1.2 *U*_eq_(C,*N*) or 1.5 *U*_eq_(methyl C). The H of the methyl group were statistically disordered over two positions.

### Crystal data

**Table:**

C_10_H_13_NO_2_	*F*(000) = 384
*M**_r_* = 179.21	*D*_x_ = 1.219 Mg m^−^^3^
Monoclinic, *P*2_1_/*c*	Mo *K*α radiation, λ = 0.71073 Å
Hall symbol: -P 2ybc	Cell parameters from 1622 reflections
*a* = 9.9206 (13) Å	θ = 3.0–26.2°
*b* = 8.7861 (11) Å	µ = 0.09 mm^−^^1^
*c* = 11.4943 (16) Å	*T* = 298 K
β = 102.998 (1)°	Block, colorless
*V* = 976.2 (2) Å^3^	0.43 × 0.38 × 0.20 mm
*Z* = 4	

### Data collection

**Table:**

Bruker SMART CCD diffractometer	1713 independent reflections
Radiation source: fine-focus sealed tube	1196 reflections with *I* > 2σ(*I*)
graphite	*R*_int_ = 0.027
φ and ω scans	θ_max_ = 25.0°, θ_min_ = 2.1°
Absorption correction: multi-scan (*SADABS*; Sheldrick, 2008)	*h* = −7→11
*T*_min_ = 0.961, *T*_max_ = 0.978	*k* = −9→10
4753 measured reflections	*l* = −13→13

### Refinement

**Table:**

Refinement on *F*^2^	Primary atom site location: structure-invariant direct methods
Least-squares matrix: full	Secondary atom site location: difference Fourier map
*R*[*F*^2^ > 2σ(*F*^2^)] = 0.037	Hydrogen site location: inferred from neighbouring sites
*wR*(*F*^2^) = 0.100	H-atom parameters constrained
*S* = 1.05	*w* = 1/[σ^2^(*F*_o_^2^) + (0.0387*P*)^2^ + 0.1837*P*] where *P* = (*F*_o_^2^ + 2*F*_c_^2^)/3
1713 reflections	(Δ/σ)_max_ < 0.001
119 parameters	Δρ_max_ = 0.16 e Å^−^^3^
0 restraints	Δρ_min_ = −0.14 e Å^−^^3^

### Special details

**Table:**

Geometry. All e.s.d.'s (except the e.s.d. in the dihedral angle between two l.s. planes) are estimated using the full covariance matrix. The cell e.s.d.'s are taken into account individually in the estimation of e.s.d.'s in distances, angles and torsion angles; correlations between e.s.d.'s in cell parameters are only used when they are defined by crystal symmetry. An approximate (isotropic) treatment of cell e.s.d.'s is used for estimating e.s.d.'s involving l.s. planes.
Refinement. Refinement of *F*^2^ against ALL reflections. The weighted *R*-factor *wR* and goodness of fit *S* are based on *F*^2^, conventional *R*-factors *R* are based on *F*, with *F* set to zero for negative *F*^2^. The threshold expression of *F*^2^ > σ(*F*^2^) is used only for calculating *R*-factors(gt) *etc*. and is not relevant to the choice of reflections for refinement. *R*-factors based on *F*^2^ are statistically about twice as large as those based on *F*, and *R*- factors based on ALL data will be even larger.

### Fractional atomic coordinates and isotropic or equivalent isotropic displacement parameters (Å^2^)

**Table:**

	*x*	*y*	*z*	*U*_iso_*/*U*_eq_	Occ. (<1)
N1	0.15094 (14)	0.38049 (16)	0.35383 (14)	0.0553 (4)	
H1	0.1936	0.4303	0.3086	0.066*	
O1	0.72697 (12)	0.10975 (15)	0.27285 (11)	0.0598 (4)	
H1A	0.7957	0.1586	0.3049	0.090*	
O2	−0.04072 (13)	0.25522 (17)	0.36772 (12)	0.0724 (4)	
C1	0.40477 (16)	0.18477 (19)	0.44391 (14)	0.0444 (4)	
C2	0.39022 (17)	0.09127 (19)	0.34533 (15)	0.0516 (5)	
H2	0.3057	0.0435	0.3162	0.062*	
C3	0.49703 (18)	0.0664 (2)	0.28865 (16)	0.0516 (5)	
H3	0.4843	0.0024	0.2226	0.062*	
C4	0.62273 (16)	0.13710 (18)	0.33044 (14)	0.0430 (4)	
C5	0.64007 (17)	0.23153 (18)	0.42830 (14)	0.0462 (4)	
H5	0.7244	0.2800	0.4568	0.055*	
C6	0.53229 (17)	0.25403 (19)	0.48397 (15)	0.0493 (4)	
H6	0.5455	0.3175	0.5503	0.059*	
C7	0.28890 (18)	0.2102 (2)	0.50690 (16)	0.0542 (5)	
H7A	0.3257	0.2034	0.5924	0.065*	
H7B	0.2210	0.1299	0.4845	0.065*	
C8	0.21757 (18)	0.3629 (2)	0.47851 (17)	0.0562 (5)	
H8A	0.1488	0.3745	0.5260	0.067*	
H8B	0.2854	0.4434	0.5012	0.067*	
C9	0.02736 (18)	0.3236 (2)	0.30588 (17)	0.0545 (5)	
C10	−0.0253 (2)	0.3440 (3)	0.17442 (18)	0.0790 (6)	
H10A	0.0416	0.3994	0.1425	0.119*	0.50
H10B	−0.1108	0.3995	0.1597	0.119*	0.50
H10C	−0.0404	0.2461	0.1366	0.119*	0.50
H10D	−0.1146	0.2973	0.1501	0.119*	0.50
H10E	0.0377	0.2972	0.1329	0.119*	0.50
H10F	−0.0326	0.4506	0.1559	0.119*	0.50

### Atomic displacement parameters (Å^2^)

**Table:**

	*U*^11^	*U*^22^	*U*^33^	*U*^12^	*U*^13^	*U*^23^
N1	0.0394 (8)	0.0592 (9)	0.0710 (10)	0.0001 (7)	0.0200 (7)	0.0143 (8)
O1	0.0472 (7)	0.0672 (9)	0.0692 (9)	−0.0047 (6)	0.0223 (6)	−0.0153 (6)
O2	0.0500 (8)	0.0981 (11)	0.0736 (9)	−0.0201 (7)	0.0232 (7)	0.0017 (8)
C1	0.0399 (9)	0.0478 (9)	0.0461 (10)	0.0014 (8)	0.0112 (7)	0.0084 (8)
C2	0.0393 (10)	0.0572 (11)	0.0565 (11)	−0.0115 (8)	0.0069 (8)	0.0006 (9)
C3	0.0503 (10)	0.0547 (11)	0.0492 (10)	−0.0075 (9)	0.0102 (8)	−0.0085 (8)
C4	0.0383 (9)	0.0445 (9)	0.0473 (10)	0.0017 (7)	0.0119 (7)	0.0022 (7)
C5	0.0348 (9)	0.0495 (10)	0.0521 (10)	−0.0034 (7)	0.0050 (7)	−0.0044 (8)
C6	0.0450 (10)	0.0542 (11)	0.0484 (10)	−0.0013 (8)	0.0096 (8)	−0.0073 (8)
C7	0.0475 (10)	0.0627 (12)	0.0545 (11)	−0.0009 (9)	0.0157 (8)	0.0079 (9)
C8	0.0439 (10)	0.0601 (12)	0.0682 (12)	−0.0054 (9)	0.0201 (9)	−0.0046 (9)
C9	0.0422 (10)	0.0601 (11)	0.0651 (12)	0.0080 (9)	0.0202 (9)	0.0039 (9)
C10	0.0602 (13)	0.1059 (18)	0.0711 (14)	0.0112 (12)	0.0150 (11)	0.0090 (13)

### Geometric parameters (Å, °)

**Table:**

N1—C9	1.324 (2)	C5—H5	0.9300
N1—C8	1.445 (2)	C6—H6	0.9300
N1—H1	0.8600	C7—C8	1.518 (2)
O1—C4	1.3686 (19)	C7—H7A	0.9700
O1—H1A	0.8200	C7—H7B	0.9700
O2—C9	1.240 (2)	C8—H8A	0.9700
C1—C2	1.381 (2)	C8—H8B	0.9700
C1—C6	1.386 (2)	C9—C10	1.494 (3)
C1—C7	1.507 (2)	C10—H10A	0.9600
C2—C3	1.380 (2)	C10—H10B	0.9600
C2—H2	0.9300	C10—H10C	0.9600
C3—C4	1.379 (2)	C10—H10D	0.9600
C3—H3	0.9300	C10—H10E	0.9600
C4—C5	1.377 (2)	C10—H10F	0.9600
C5—C6	1.378 (2)		
			
C9—N1—C8	123.14 (15)	N1—C8—H8A	108.9
C9—N1—H1	118.4	C7—C8—H8A	108.9
C8—N1—H1	118.4	N1—C8—H8B	108.9
C4—O1—H1A	109.5	C7—C8—H8B	108.9
C2—C1—C6	116.94 (15)	H8A—C8—H8B	107.7
C2—C1—C7	122.18 (15)	O2—C9—N1	121.13 (17)
C6—C1—C7	120.88 (15)	O2—C9—C10	121.75 (17)
C3—C2—C1	122.12 (16)	N1—C9—C10	117.12 (17)
C3—C2—H2	118.9	C9—C10—H10A	109.5
C1—C2—H2	118.9	C9—C10—H10B	109.5
C4—C3—C2	119.64 (16)	H10A—C10—H10B	109.5
C4—C3—H3	120.2	C9—C10—H10C	109.5
C2—C3—H3	120.2	H10A—C10—H10C	109.5
O1—C4—C5	122.12 (14)	H10B—C10—H10C	109.5
O1—C4—C3	118.36 (15)	C9—C10—H10D	109.5
C5—C4—C3	119.52 (15)	H10A—C10—H10D	141.1
C4—C5—C6	119.89 (15)	H10B—C10—H10D	56.3
C4—C5—H5	120.1	H10C—C10—H10D	56.3
C6—C5—H5	120.1	C9—C10—H10E	109.5
C5—C6—C1	121.89 (16)	H10A—C10—H10E	56.3
C5—C6—H6	119.1	H10B—C10—H10E	141.1
C1—C6—H6	119.1	H10C—C10—H10E	56.3
C1—C7—C8	113.41 (14)	H10D—C10—H10E	109.5
C1—C7—H7A	108.9	C9—C10—H10F	109.5
C8—C7—H7A	108.9	H10A—C10—H10F	56.3
C1—C7—H7B	108.9	H10B—C10—H10F	56.3
C8—C7—H7B	108.9	H10C—C10—H10F	141.1
H7A—C7—H7B	107.7	H10D—C10—H10F	109.5
N1—C8—C7	113.25 (15)	H10E—C10—H10F	109.5

### Hydrogen-bond geometry (Å, °)

**Table:**

*D*—H···*A*	*D*—H	H···*A*	*D*···*A*	*D*—H···*A*
N1—H1···O1^i^	0.86	2.08	2.9048 (18)	161
O1—H1A···O2^ii^	0.82	1.83	2.6464 (17)	174

Symmetry codes: (i) −*x*+1, *y*+1/2, −*z*+1/2; (ii) *x*+1, *y*, *z*.

## References

[bb1] Allen, F. H., Kennard, O., Watson, D. G., Brammer, L., Orpen, A. G. & Taylor, R. (1987). *J. Chem. Soc. Perkin Trans. 2*, pp. S1–19.

[bb2] Bernstein, J., Davis, R. E., Shimoni, L. & Chang, N.-L. (1995). *Angew. Chem. Int. Ed. Engl.* **34**, 1555–1573.

[bb3] Bruker (1998). *SMART* Bruker AXS Inc., Madison, Wisconsin, USA.

[bb4] Bruker (1999). *SAINT* Bruker AXS Inc., Madison, Wisconsin, USA.

[bb5] Burnett, M. N. & Johnson, C. K. (1996). *ORTEPIII* Report ORNL-6895. Oak Ridge National Laboratory, Tennessee, USA.

[bb6] Chai, Y., Wan, Z.-L., Guo, H.-Y. & Liu, M.-L. (2009). *Acta Cryst.* E**65**, o282. 10.1107/S1600536809000634 PMC2968237 21581895

[bb7] Etter, M. C. (1990). *Acc. Chem. Res.* **23**, 120–126.

[bb8] Farrugia, L. J. (1997). *J. Appl. Cryst.* **30**, 565.

[bb9] Garcez, W. S., Martins, D. & Garcez, F. R. (2000). *J. Agric. Food Chem.* **48**, 3662–3665. 10.1021/jf991146o 10956166

[bb10] Montedoro, G., Servili, M. & Baldioli, M. (1993). *J. Agric. Food Chem.* **41**, 2228–2234. 10.1021/jf9806210 10563841

[bb11] Pearce, L., Prout, C. K. & Watkin, D. J. (2000). *CAMERON* Chemical Crystallography Laboratory, University of Oxford, England.

[bb14] Sheldrick, G. M. (2008*a*). *SADABS.* University of Göttingen, Germany.

[bb12] Sheldrick, G. M. (2008*b*). *Acta Cryst.* A**64**, 112–122. 10.1107/S0108767307043930 18156677

[bb13] Song, W.-L., Wang, D., Li, X.-H. & Wang, D.-C. (2008). *Acta Cryst.* E**64**, o785. 10.1107/S1600536808005989 PMC2961301 21202278

